# Using baited remote underwater videos (BRUVs) to characterize chondrichthyan communities in a global biodiversity hotspot

**DOI:** 10.1371/journal.pone.0225859

**Published:** 2019-12-04

**Authors:** Geoffrey J. Osgood, Meaghen E. McCord, Julia K. Baum

**Affiliations:** 1 Department of Biology, University of Victoria, Victoria, British Columbia, Canada; 2 South African Shark Conservancy (SASC), Hermanus, Western Cape, South Africa; Institut de Recherche Pour le Developpement, FRANCE

## Abstract

Threatened chondrichthyan diversity is high in developing countries where scarce resources, limited data, and minimal stakeholder support often render conservation efforts challenging. As such, data on many species, including many evolutionarily distinct endemics, is poor in these countries and their conservation status and habitat needs remain uncertain. Here, we used baited remote underwater videos (BRUVs; n = 419) conducted at 167 sites over two years to assess the frequency of occurrence (FO), relative abundance, diversity, and structure of chondrichthyan assemblages in one of the world’s chondrichthyan biodiversity and endemism hotspots, South Africa. We compared chondrichthyan assemblages across three habitat types, and between unprotected and protected areas (a small marine protected area [MPA] and a larger, seasonal whale sanctuary). Although in total we observed 18 chondrichthyan species (11 families), over half of all observations were of just two species from the same family of mesopredatory endemic catsharks; only 8.8% were larger shark species. These mesopredatory species do not appear to be threatened, but some skates and larger shark species, including some endemics, were much rarer. Overall chondrichthyan FO was high (81% of all BRUVs); FO was higher in kelp (100% of BRUVS) and reef (93%) sites than at sites in sandy habitat (63%), which had a distinct chondrichthyan community. Independent of habitat, the chondrichthyan community did not relate strongly to protection. Because sites with kelp and reef habitat were rare in the whale sanctuary, this protected area had a lower chondrichthyan FO (67% of BRUVs) than either unprotected sites (81%) or those in the small MPA (98%), as well as having lower chondrichthyan relative abundance and species richness. Our study provides evidence of the importance of distinct habitat types to different chondrichthyan species, and suggests that even small MPAs can protect critical habitats, such that they may provide safe refuge for endemic species as anthropogenic pressures increase.

## Introduction

Threats from overfishing, habitat degradation, and pollution are heightened for many chondrichthyan (sharks, skates, rays, and chimaeras) species because their life history characteristics, including a late age of sexual maturity and small litter sizes, correspond to slow population growth rates [1,2]. These threats have already resulted in significant chondrichthyan population declines in many regions, especially for coastal species [1,3]. For most species, however, a paucity of data hinders management by stock assessment or assessment of their conservation status [1]. Marine protected areas (MPAs), where fishing, and often other human activities, are either restricted or illegal, have been employed to promote chondrichthyan conservation in some regions [4], with some success, particularly on coral reef systems [5–7]. It is recognized, however, that additional tools to effectively conserve shark populations are likely required, and that conservation measures would benefit from species-specific biological data [8].

In many developing countries chondrichthyan biodiversity and endemism are high, but a lack of resources to study and manage the species means their conservation status remains unknown [7, 8]. Typically the data required to conduct population (stock) assessments is lacking for chondrichthyans, meaning spatial protections may instead be relied upon for their conservation, especially when multiple threats, from habitat destruction to fishing pressure, need to be managed [9–11]. However, MPA design also requires species-specific knowledge, as even related chondrichthyans show considerable variation in residency patterns and in preferred depths and habitat types [12,13]. Since research effort is often concentrated on a few charismatic chondrichthyan species, many species, particularly endemics, remain poorly understood globally with little information on their populations [1,14]. Managers require this information to assess what management measures may be appropriate for a broad range of taxa, including both mesopredators and more mobile apex predators, and to decide if MPAs protect sufficient critical habitat [15,16]. Therefore, there is a need to assess the diversity and conservation status of overlooked but threatened chondrichthyan species and to relate that diversity to habitat characteristics and current management schemes in developing countries. Local knowledge in these hotspots scales is critical if global chondrichthyan diversity is to be conserved.

South Africa, a global biodiversity hotspot with high chondrichthyan endemism (~30% species), exemplifies the challenges associated with chondrichthyan conservation. Although shark and ray species have significant cultural and natural heritage value in the region [9,17], they are both threatened by multiple stressors, including coastal development, pollution, and heavy fishing pressure [17–20], and poorly studied, such that knowledge of the abundance and distribution is limited for most species. Although MPAs now cover almost a quarter of South Africa’s coast, the extent to which they protect biodiversity from both fishing and other threats is still unknown, as most were established without clear management objectives or ecological information [21,22]. Additionally, corruption, poverty and stakeholder conflict limit the success of many conservation measures in the country [23].

We focus herein on the sub-temperate Cape Whale Coast in the Western Cape of South Africa, a stretch of coastline that is home to at least sixty chondrichthyan species, many of which are endemic (e.g. the spotted gully shark *Triakis megalopterus*, pyjama catshark *Poroderma africanum*, leopard catshark *P*. *pantherinum*, dark shyshark *Haploblepharus pictus*, and puffadder shyshark *H*. *edwardsii*). The few studies that have investigated the biology of these species suggest that these chondrichthyans use a diversity of coastal habitats, from sandy bays to kelp forests and temperate rocky reefs [24,25], although knowledge of species-specific habitat preferences is limited.

The conservation status of chondrichthyans on the Cape Whale Coast, and the role of MPAs in protecting the region’s chondrichthyan biodiversity is uncertain. The region currently includes two small protected areas: the small Betty’s Bay MPA (20 km^2^), which has year-round prohibition of boat-based activity (albeit with shore-angling allowed) and Walker Bay Whale Sanctuary, a larger, seasonal MPA located in the inner 113 km^2^ of Walker Bay. Betty’s Bay MPA was established initially in 1973 to protect South African abalone *Haliotis midae* and linefish stocks, and is now important for the conservation of Endangered African penguin *Spheniscus demersus* [26]. The Walker Bay Whale Sanctuary was established in 2001 under South Africa’s *Marine Living Resources Act* (Act No. 18 of 1998) to protect the southern right whale *Eubalaena australis*, and is only in effect during their calving season (July to December), when all vessels except permitted whale watching boats are prohibited. To date, only a year-long preliminary survey of the fishes and benthic invertebrates in Betty’s Bay has been done, showing weak to no effects of protection [27].

Overall, the Cape Whale Coast is heavily impacted by fishing pressure and coastal development, with unknown consequences for the area’s chondrichthyans. A century-old line fish fishery collapsed in the late-1990s [28], and considerable small-scale fishing and commercial line and seine fishing continues to occur [20,29], and is common around the two MPAs when pilchard *Sardinops sagax* and snoek *Thyrsites atun* are running. Larger sharks, including the soupfin shark *Galeorhinus galeus*, common smoothhound shark *Mustelus mustelus*, and bronze whaler *Carcharhinus brachyurus* are targeted in commercial and recreational fisheries (100–400 t per year), whereas most smaller mesopredatory chondrichthyans are caught incidentally (1–10 t per year) in linefish and lobster fisheries or by recreational anglers, most of which is catch-and-release [20]. Small, endemic catsharks are taken as bycatch in small shore-based recreational and subsistence fisheries targeting valuable reef fish and large sharks within the Walker Bay Whale Sanctuary and the Betty’s Bay MPA (M. McCord, pers. obs.). Although only a few catsharks are retained for local consumption and illegal sale, poor catch and release practices and improper handling likely result in high post-release mortality rates of those sharks that are released (M McCord, pers. obs). Few data on the population trends of these endemic species exist, and data required for stock assessments exist for less than 10% of the chondrichthyans found in the region [20]. Thus, the impacts of incidental fishing on South Africa’s endemic sharks remain unknown. Coastal development and pollution also threaten these endemic species to an unknown extent, particularly *H*. *edwardsii*, which is currently listed as Near Threatened on the IUCN Red list [30].

Given an overwhelming paucity of data to support chondrichthyan management and conservation in South Africa, this study employed baited remote underwater video (BRUV), a common, non-invasive technique for monitoring mobile and rare species [12,31], to provide first insights into the ecology of local chondrichthyan species–with a focus on endemic sharks—in relation to habitat and protected areas, in this important biodiversity hotspot. We quantified the abundance and diversity (in terms of species, higher level taxonomy, and trophic levels) of chondrichthyans, and characterized their community structure, across three habitat types inside and outside of each of the two protected areas, and then modelled how these factors varied amongst habitats and across protection levels. Finally, we evaluated if habitat differences across protection zones accounted for differences in diversity in order to assess the future potential of these MPAs for conserving South Africa’s rich and diverse chondrichthyan heritage. We hypothesized that mesopredatory endemic chondrichthyans would dominate the abundance and diversity of the community in all habitats and protection zones, being released from predation due to likely declines of larger sharks in fisheries. We further hypothesized that chondrichthyan diversity, relative abundance, and community composition would not vary with protection, but instead be driven primarily by habitat type as neither MPA is no-take, both are small, and both were designed based on the ecological needs of other taxa.

## Methods and materials

### Sampling design

**Fig 1:**
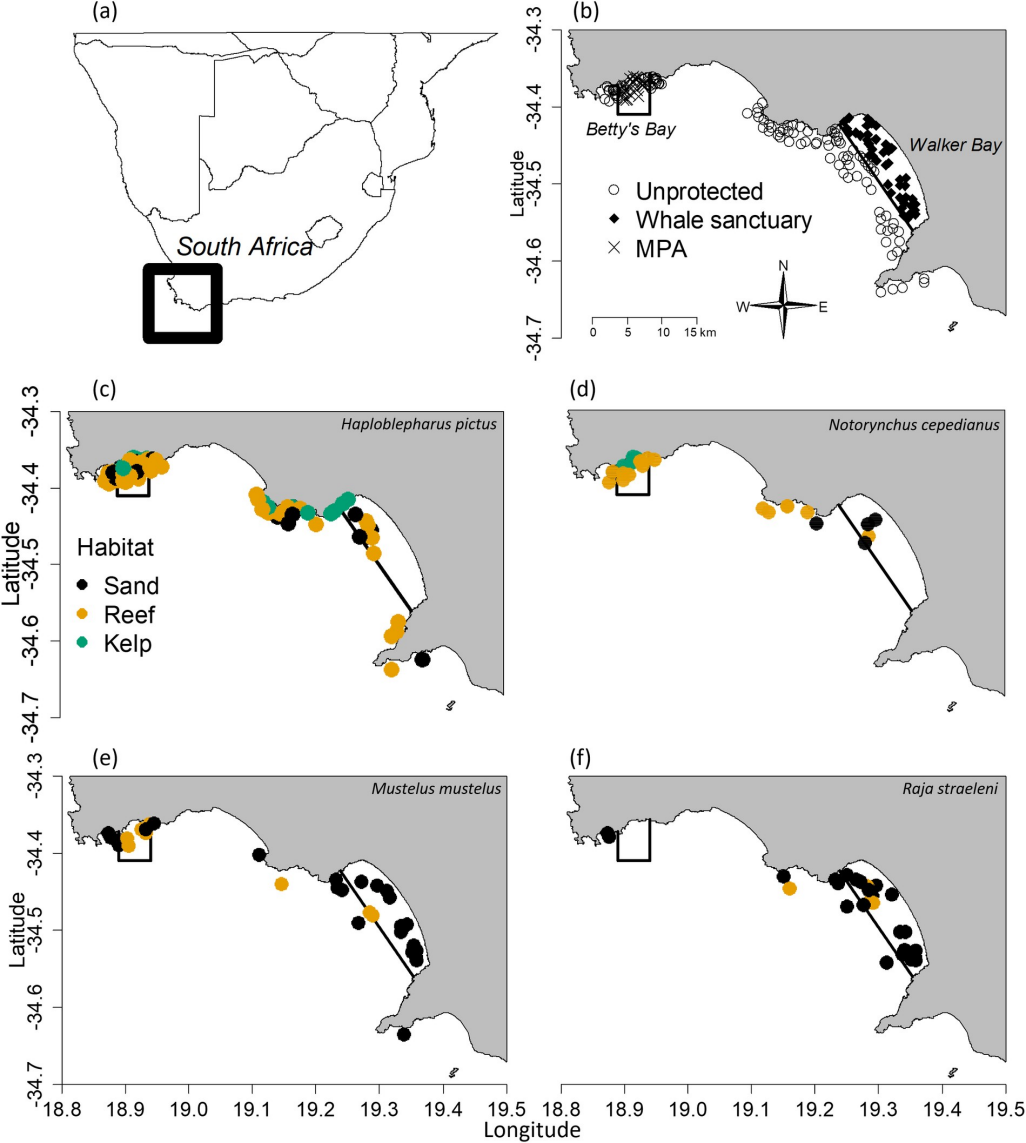
Maps of sampling sites showing protection levels and locations and habitats of commonly observed species. (a) The study area within southern Africa (black circle); (b-f) maps of the study area showing (b) the two protected areas (Walker Bay Whale Sanctuary; Betty’s Bay MPA) with all BRUV sites categorized by protection level; observations of five representative species categorized by habitat type: (c) dark shyshark *Haploblepharus pictus* (most abundant shark); (d) broadnose sevengill shark *Notorynchus cepedianus* (most abundant high trophic level shark); (e) common smooth-hound shark *Mustelus mustelus* (most abundant triakid); and (f) biscuit skates *Raja straeleni* (most abundant endemic batoid). Legend for habitat colour in (c) applies to (d-f).

Over a two-year period (July 2016—July 2018), in both winter-spring (June-November) and summer-fall (December-May), we deployed a total of 419 BRUVs at 167 sites along the South African coastline in two regions: Betty’s Bay and Walker Bay (Fig 1B, S1 Table). We made efforts to sample each site once in each season, but constraints due to weather and equipment sometimes prevented this. At least some sites in each region and level of protection were sampled in each season. Ultimately, a total of 233 BRUV drops occurred in the summer-fall and 186 BRUV drops in the winter-spring (S1 Table). A BRUV drop represents a replicate at a site, and between one to five (mean = 2.5) drops occurred per site. Sites were randomly placed stratified among the Walker Bay Whale Sanctuary (n = 40 sites total, 109 total drops) and Betty’s Bay MPA (n = 29 sites total, 85 total drops), and areas outside each in Walker Bay (n = 69, 131 total drops) and Betty’s Bay (n = 29, 94 total drops) (Fig 1A, S4 Table). We note that the sites sampled in Betty’s Bay were the same as previously sampled by colleagues [27]. Sampling sites were 500 m apart except for within the Betty’s Bay MPA where, due to the smaller area sampled, sites were at minimum 100–200 m apart. Whenever possible, sites closer than 500 m apart were not sampled on the same day. The depths of sampled sites ranged between 3 m and 55 m (mean = 25.3 m, standard deviation (SD) = 12.2 m). We made efforts to sample across all habitat types (kelp forest, sand and rocky reef) within each region and protected area, but relied on random sampling to reflect the habitat frequency within each region, since there are no detailed data on distributions of habitat in either region.

### Baited remote underwater video (BRUV) design and analysis

Each BRUV rig was composed of a mild-steel cross-shaped base with a bait canister and camera set 110 cm apart. The bait canister and camera were raised 20–30 cm off the rig’s bottom by bending the ends of steel arms 90° vertically. One meter of stainless steel chain attached the rig to a rope leading to a surface buoy. One kilogram of chopped, defrosted sardine (*Sardinops sagax*) was placed into each bait canister. We used GoPro® cameras (Hero 1, Hero 2, Hero 3 Silver Edition, Hero+) set to 720p.

All BRUVs were deployed between 8:00 and 15:00, at least half an hour after sunrise and three hours before sunset. Water visibility varied between 0.5 m and approximately 20 m at each site. Target deployment time was 67 minutes, allowing bait to disperse and leaving 60 minutes of footage to analyze, but due to field conditions and camera failure, actual recording time ranged from 20.7 minutes to 103.2 minutes (mean = 62.7, SD = 10.7).

For each BRUV at each site, we recorded the percentage of BRUV drops on which a chondrichthyan occurred (frequency of occurrence or FO), and the maximum number of individuals observed together for one species at any one time on the entire video (MaxN; following [32]). MaxN is a commonly used conservative measure of species’ relative abundance in BRUV analyses because it avoids double-counting [12,32]. The shyshark *H*. *edwardsii* was distinguished from the closely related *H*. *pictus* using the former’s broad head and distinct dark-margined dorsal saddles, often with orange-yellow coloration inside. From each BRUV, we recorded the dominant (>50% cover) habitat type (sand, rocky reef, kelp) and visibility in broad categories, using the distance to the bait canister as a guide (1 = <1 m; 2 = 1–5 m; 3 = 5–10 m; 4 = >10 m). We recorded depth and sea surface temperature (SST) using a HDS-8m Gen2 Lowrance chartplotter for deployments in Betty’s Bay. In Walker Bay, we recorded depth using one meter markings on the BRUV rope and daily SST as the GOES-POES 5-km Blended SST from PacIOOS (http://www.pacioos.hawaii.edu/voyager/#). For each species we observed, we retrieved trophic levels from FishBase, which were determined from mean trophic level of diet items.

All observations of live animals were authorized by the University of Victoria Animal Care Committee (AUP 2016-032(1)) for this study and conducted under the authority of a joint research permit issued by the South African Department of Agriculture, Forestry and Fisheries and Department of Environmental Affairs: Oceans and Coasts Branch (RES2017-31 and RES2018-59).

We used available catch data from three sources to compare with the species composition found on the BRUVs. We obtained data on chondrichthyan recreational catches collected by volunteer anglers to the Cooperative Fish Tagging Project of South Africa’s Oceanographic Research Institute (ORI) since 2012 in Betty’s Bay. We also obtained data from four Rock and Surf Super Pro League (RASSPL) recreational angling competitions over the last ten years in the Betty’s Bay MPA. Finally, we used the South African Shark Conservancy’s (SASC) database of chondrichthyans tagged during biological sampling from both shore and boat handline fishing in the Walker Bay region since 2010. These data were the only available, but have the following caveats: 1) anglers participating in the ORI tagging project typically do not tag or record endemic sharks due to their perceived lack of importance; 2) RASSPL anglers generally only record larger sharks; and 3) the SASC dataset does not include the Betty’s Bay MPA. Therefore these analyses were qualitative due to sampling biases and associated analytical constraints.

### Statistical analysis

We analyzed the effects of protection and habitat on chondrichthyan FO, relative abundance (summed MaxN across all species), and species richness using generalized linear mixed models (GLMM), with a binomial error distribution for FO and a Poisson error distribution for the latter two. Although we initially considered a negative binomial error structure for the relative abundance and species richness models, residual versus fitted plots and likelihood ratio tests revealed that it did not provide a better fit and we therefore used the Poisson distribution for each. For each model, we included region (Walker Bay versus Betty’s Bay), protection (inside or outside MPA), the interaction of region and protection, habitat type (sand, temperate rocky reef, kelp forest), SST, depth, visibility, year, and seasonality (sine and cosine of study Julian day divided by 365) as fixed effects, and site as a random effect. We also included duration of the video as an offset. We included the interaction of region and protection to account for differing effects of the two MPAs (one seasonal, one allowing shore fishing); we removed the interaction if it was not significant. We used a likelihood ratio test to determine if protection and habitat, and their interaction, significantly improved the likelihood of each GLMM. To ascertain the significance of other variables, and to determine which individual levels of protection and habitat were significant, we used a Wald's Z test on the coefficients of each GLMM. We also examined residual versus fitted plots to check for major model misspecification.

We then repeated the FO and relative abundance analyses described above on three species groups of interest: the endemic mesopredatory catsharks (the most abundant sharks in the region); larger-bodied sharks as a group (broadnose sevengill shark *Notorhyncus cepedianus*, *T*. *megalopterus*, *G*. *galeus*, *M*. *mustelus*, *C*. *brachyurus*, hammerhead shark *Sphyrna* sp.); and batoids. As the most batoids we observed at once was two, we focus only on FO GLMMs for the batoids.

Next, to assess how protection and habitat influenced chondrichthyan community structure, we used two complementary multivariate statistical techniques. First, we constructed multivariate regression trees (MRT) using the R package *mvpart* [33]. We used this clustering technique to evaluate which variable (protection, habitat, depth, season, SST, visibility) best differentiated chondrichthyan communities (based on their MaxN values) at different sites. We then calculated Dufrêne-Legendre Indicator (DLI) values to determine which species served as indicators of each cluster identified in the MRT. Significance of DLI was determined with a permutation test with 1000 permutations and we deemed species with a DLI > 0.15 to be important indicators. Second, we implemented a recently developed ordination technique (*boral* package, [34]) that enabled us to visualize the variation in chondrichthyan community composition across sites, to identify individual chondrichthyan species that distinguished sites, and to verify the clusters identified by the MRT. To construct the ordination, the *boral* package uses Bayesian latent variable models in which the ordination axes represent the two most important latent variables fitted to the community at each site [34]. The corresponding latent variable coefficients, which represent the contribution of each species to that axis, are plotted with their scores to make a biplot. We included a site-level random effect to focus on community composition rather than variation in abundance, since this allows for the fact that communities at different sites with identical species compositions could have different abundances. We also included total video duration as an offset. Bayesian latent variable models are appropriate for multivariate data with correlated response variables and a strong mean-variance relationship, such as our count and presence-absence data, and are preferred to distance-based analyses (eg. PCA, MDS), which have low power to detect differences except for species with high variance, even after transformations, and harder to evaluate methodological assumptions [34,35].

To account for potential spatial autocorrelation between sites, we calculated spatial eigenfunctions using distance-based Moran's eigenvector maps, staggered for Walker Bay and Betty’s Bay, using the R function create.dbMEM.model in the package *adespatial* [36]. We kept eigenvectors corresponding to positive spatial autocorrelation for use in each of our multivariate and univariate models, besides the MRT.

We verified complete sampling of the chondrichthyan community in our BRUVs by constructing a species accumulation curve for the data in each of the three protection levels, randomizing the order of samples and calculating an average curve from 999 permutations with the specaccum function of the package *vegan* [37].

We conducted all analyses in R version 3.5.0 [38]. We used the package glmmADMB to run the generalized linear mixed models [39] and the function *indval* in the package labdsv to calculate DLI values [40]. Data are available online (https://zenodo.org/badge/latestdoi/194944885). All the code for the analyses will be available in a GitHub repository at a URL made available upon manuscript acceptance.

## Results

**Fig 2:**
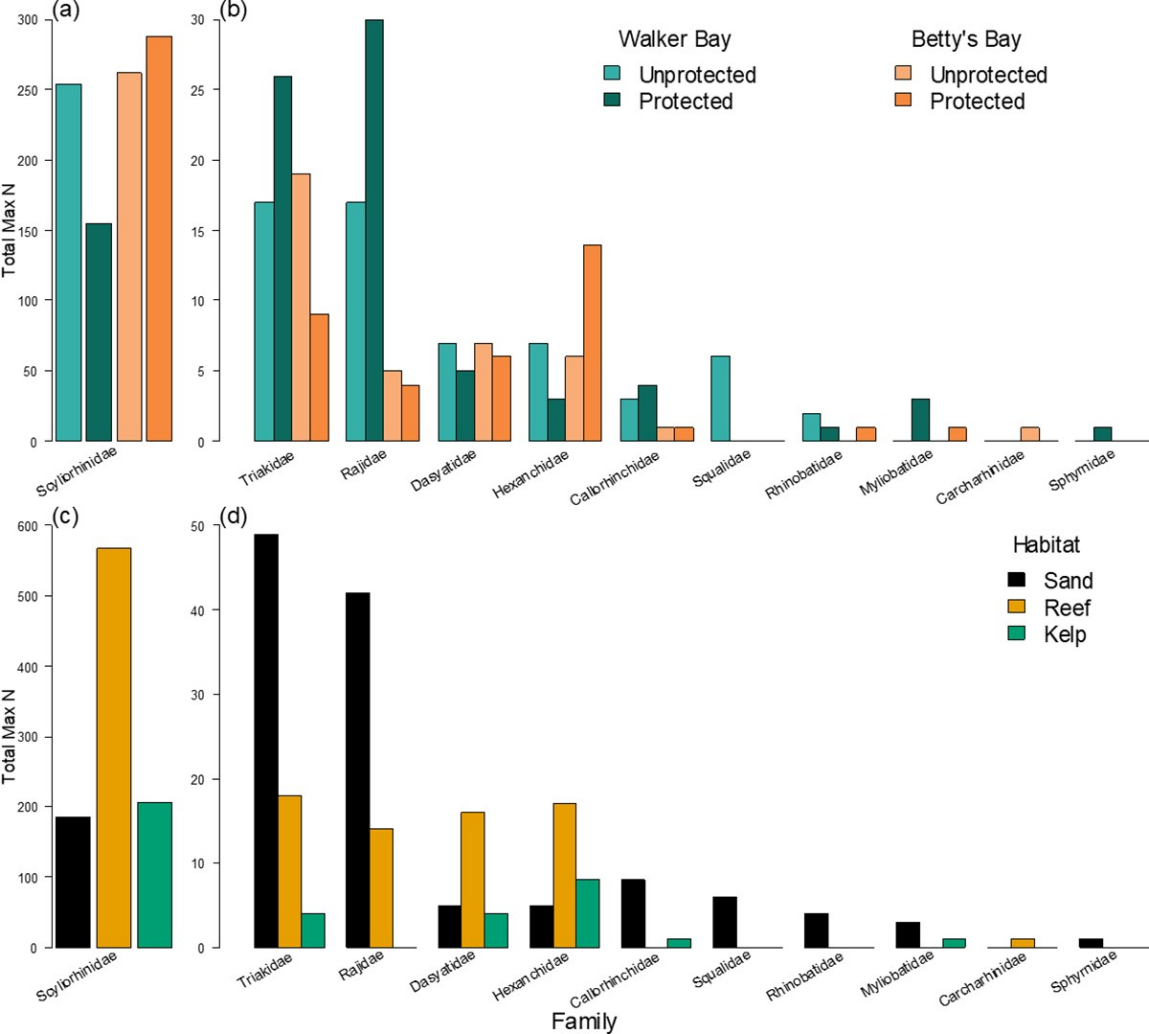
**Max N summed for all chondrichthyans species in each family over all BRUVs by (a,b) protection level in each region and (c,d) by habitat.** (a, c) The most commonly observed chondrichthyan family, the scyliorhinid catsharks. (b, d) The remaining chondrichthyan families. Note the different scales on the y-axes.

Overall, we counted 1166 chondrichthyans on 419 videos. These included 18 chondrichthyan species from 11 families: 14 species of shark, 3 species of batoid, and one species of holocephalan (Table 1). Half of the observed chondrichthyan species, spanning five families, are endemic to southern Africa (Table 1). Despite this taxonomic and phylogenetic diversity, two species (*H*. *pictus* and *P*. *poroderma*) and one family (Scyliorhinidae) of mesopredatory sharks dominated our observations, accounting for 53% and 82% of all chondrichthyans, respectively (Table 1, Fig 2). These two species occurred throughout each region and in all habitats, although most were observed within the Betty’s Bay MPA (Table 1, Fig 1C). Only 8.8% of the counted chondrichthyans were larger shark species, and of those *M*. *mustelus* was the most frequently observed (10% of BRUVs), particularly in the MPAs (Fig 1E), where it collectively occurred on 17% of sand sites. We also commonly observed *N*. *cepedianus* in the Betty’s Bay MPA (15% of BRUVs, Fig 1D) and biscuit skates *Raja straeleni* in the Walker Bay Whale Sanctuary (18% of BRUVs, Fig 1F). The rarest species were *C*. *brachyurus*, *Sphyrna* sp., and shortnose spurdog *Squalus megalops*, each observed on only one occasion. The mean trophic level (+/- SD) of all counted individuals was 4.20 +/- 0.35 due to the high abundance of mesopredatory catsharks (trophic level 3.6 to 4.2) (Table 1). One third of the observed species are listed as data deficient on the IUCN Red List, while four species (22%) are threatened (Table 1). Species accumulation curves in each area, and overall, reached an asymptote, indicating complete sampling of the chondrichthyan community (S1 Fig).

**Table 1:** Summary of the taxonomy, endemism, IUCN Red List status, population trend on the IUCN Red List (Version 2019–2), trophic level, and relative abundance (FO, MaxN)^a^ of the chondrichthyan species observed on BRUVs, ordered from highest to lowest FO within each taxonomic group (Sharks, Batoidea, Holocephali).

							Walker Bay				Betty’s Bay			
							Unprotected sites		Whale Sanctuary		Unprotected sites		MPA	
Species	Common name (abbreviation)	Family	Endemic (Y/N)	Trophic level^*b*^	IUCN^*a*^	Population trend	FO	Max N	FO	Max N	FO	Max N	FO	Max N
Sharks														
*Haploblepharus pictus*	Dark shyshark (DS)	Scyliorhinidae	Y	4.2	LC	Unknown	0.34	1.51	0.14	1.60	0.81	1.39	0.89	1.72
*Poroderma africanum*	Pyjama catshark (PJ)	Scyliorhinidae	Y	3.6	NT	Unknown	0.27	1.60	0.22	2.75	0.54	1.59	0.61	1.65
*Haploblepharus edwardsii*	Puffadder shyshark (PA)	Scyliorhinidae	Y	3.8	NT	Unknown	0.21	3.11	0.15	2.25	0.21	1.20	0.16	1.36
*Poroderma pantherinum*	Leopard catshark (LP)	Scyliorhinidae	Y	4.1	DD	Unknown	0.21	1.21	0.08	1.33	0.30	1.43	0.42	1.22
*Mustelus mustelus*	Common smooth-hound (CS)	Triakidae	N	3.8	VU	Decreasing	0.08	1.00	0.15	1.25	0.11	1.30	0.06	1.00
*Halaelurus natalensis*	Tiger catshark (TC)	Scyliorhinidae	Y	4.2	DD	Unknown	0.06	1.50	0.13	1.21	0.09	1.38	0.06	1.60
*Notorynchus cepedianus*	Broadnose sevengill (BG)	Hexanchidae	N	4.7	DD	Unknown	0.05	1.00	0.03	1.00	0.05	1.20	0.15	1.08
*Galeorhinus galeus*	Soupfin shark (SF)	Triakidae	N	4.3	VU	Decreasing	0.05	1.00	0.04	1.00	0.04	1.00	0.02	1.00
*Triakis megalopterus*	Spotted-gully shark (SG)	Triakidae	Y	4.0	NT	Unknown	0.00	0.00	0.02	1.00	0.02	1.00	0.02	1.00
*Squalus megalops*	Shortnose spurdog (SD)	Squalidae	N	4.3	DD	Unknown	0.01	6.00	0.00	0.00	0.00	0.00	0.00	0.00
*Carcharhinus brachyurus*	Bronze whaler (BW)	Carcharhinidae	N	4.5	NT	Unknown	0.00	0.00	0.00	0.00	0.01	1.00	0.00	0.00
*Sphyrna sp*.	Hammerhead shark (SH)	Sphyrnidae	N	4.9	VU	Decreasing	0.00	0.00	0.01	1.00	0.00	0.00	0.00	0.00
Batoidea														
*Raja straeleni*	Biscuit skate (BS)	Rajidae	Y	4.0	DD	Unknown	0.06	1.13	0.18	1.15	0.02	1.00	0.00	0.00
*Rostroraja alba*	Spearnose skate (SN)	Rajidae	N	4.4	EN	Decreasing	0.06	1.00	0.06	1.00	0.03	1.00	0.05	1.00
*Bathytoshia brevicaudata*	Short-tail stingray (SR)	Dasyatidae	N	3.9	LC	Stable	0.05	1.00	0.05	1.00	0.07	1.00	0.07	1.00
*Rhinobatos annulatus*	Lesser guitarfish (LG)	Rhinobatidae	Y	3.4	LC	Unknown	0.02	1.00	0.01	1.00	0.00	0.00	0.01	1.00
*Myliobatis aqulia*	Eagle ray (ER)	Myliobatidae	N	3.6	DD	Unknwon	0.00	0.00	0.03	1.00	0.00	0.00	0.01	1.00
Holocephali														
*Callorhinchus capensis*	St. Joseph shark (SJ)	Callorhinchidae	Y	3.5	LC	Stable	0.02	1.00	0.04	1.00	0.01	1.00	0.01	1.00

^*a*^Abbreviations: LC, least concern; NT, near threatened; VU, vulnerable; EN, endangered; DD, data deficient; FO, frequency of occurrence (ie. proportion of videos observed on); MaxN, maximum number of individuals observed per species per video averaged across sites where the species occurred.

^*b*^ Trophic levels taken from FishBase (www.fishbase.org).

The RASSPL recreational catch and SASC tagging data had a similar relative abundance of Scyliorhinidae over other chondrichthyan species in the region, comprising 76% and 93% of these records, respectively (S2 Table). In contrast, the ORI tagging database was dominated by *T*. *megalopterus* and *N*. *cepedianus* (68% and 26% of 243 records, respectively), with no records of Scyliorhinidae in its seven years. *Notorynchus cepedianus* was the second most abundantly caught species (1.6% of 1850 records) after scyliorhinids in the SASC database (none were captured from shore). However, triakids as a group were the most commonly captured after Scyliorhinidae when considering all databases (S2 Table). *Triakis megalopterus* was abundant in RASSPL (7.7% of 310 records), but less so in SASC records (0.5% of 1138 records from shore, 0.65% of 1850 total). In the SASC database, for which species-specific data is most reliable, *H*. *pictus* were the most abundantly captured chondrichthyan (46% of 1850 records). Only a few records of the brown shyshark *Haploblepharus fuscus* and ragged tooth shark *Carcharias taurus* were species not on the BRUVs (S2 Table).

### Chondrichthyan frequency of occurrence and relative abundance

We observed chondrichthyans in 81% of all BRUVs and at 88% of all sites, but FO was higher in reef and kelp sites (93% and 100% of BRUVs, respectively) than in sandy habitat (63%). Chondrichthyan FO was high in Betty’s Bay, both within the MPA (98% of BRUVs) and outside of it (95% of BRUVs), compared to Walker Bay, where it was much lower, both inside the Whale Sanctuary (67% of BRUVs) and outside it (72% of BRUVs). Across both regions, chondrichthyans were observed at 81% of BRUVs at unprotected sites. Both habitat (LRT, χ^2^ = 15.48, df = 2, p<0.001) and region (LRT, χ^2^ = 5.17, df = 1, p = 0.023) improved the FO model fit, but protection did not (LRT, χ^2^ = 0.286, df = 1, p = 0.59) (S3 Table).

**Fig 3:**
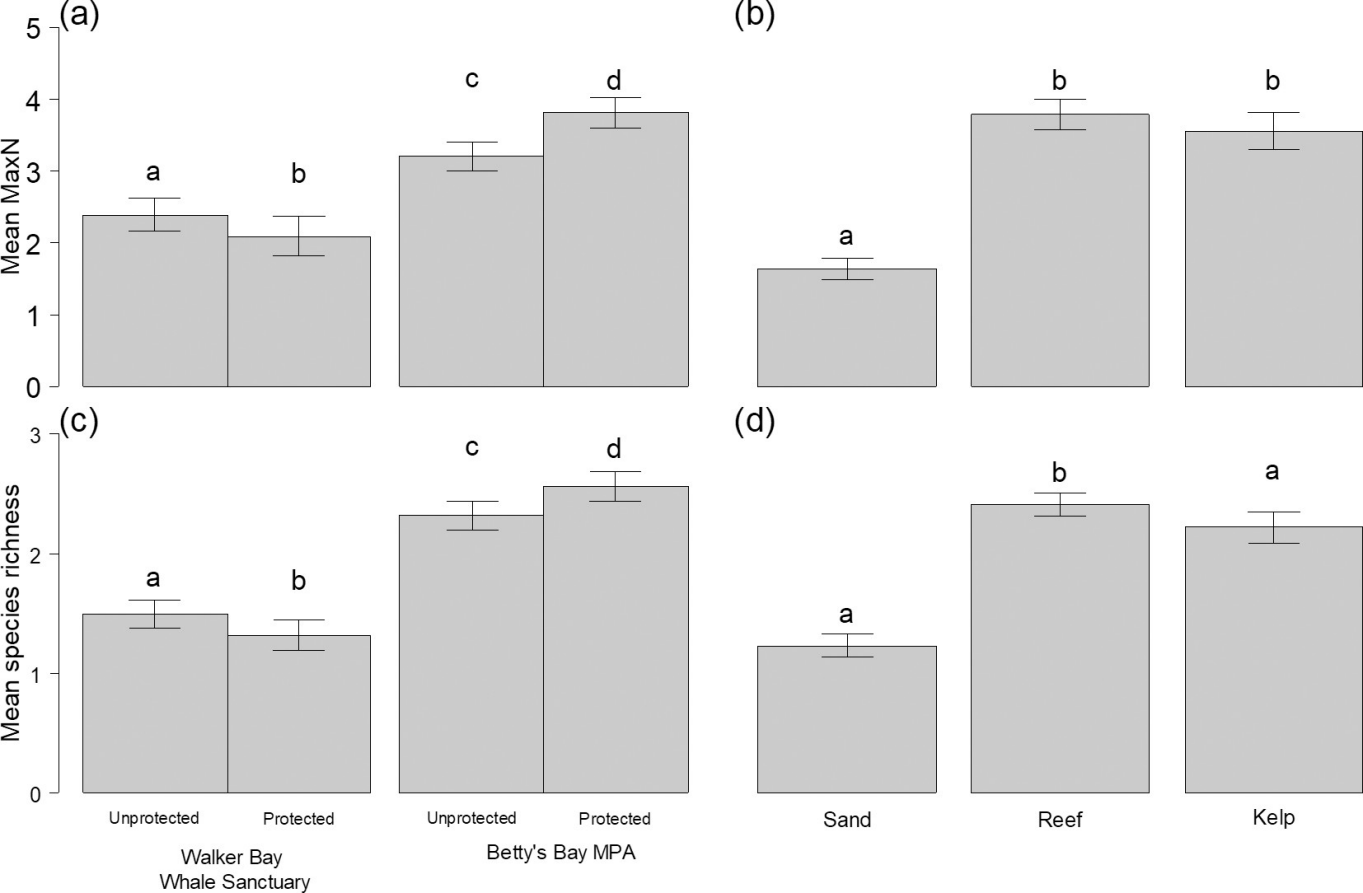
Mean chondrichthyan relative abundance and richness by protection and habitat. (a, b) Mean summed MaxN per BRUV and (c,d) mean species richness per BRUV, compared across: (a, c) protection level in each region and (b, d) habitat type. Bars are +/- SE. Comparisons with the same letter were not significantly different.

The relative abundance of chondrichthyans varied significantly by habitat (LRT, χ^2^ = 22.1, df = 2, p<0.001), with a predicted 59% and 63% more chondrichthyans observed in reef and kelp, respectively, compared to sandy habitat (Fig 3B, S3 Table). Protection had a smaller significant effect on relative abundance that varied by region (LRT, χ^2^ = 5.1, df = 1, p = 0.024): the Betty’s Bay MPA had a predicted 11% greater relative abundance of chondrichthyans compared to its adjacent unprotected sites, whereas the Walker Bay Whale Sanctuary had a predicted 50% lower relative abundance compared to its adjacent unprotected sites, based on model coefficients once other variables (region, depth, etc.) were controlled for (Fig 3A). Directly comparing the two protected areas, the Betty’s Bay MPA had a significantly greater chondrichthyan abundance per BRUV (predicted increase of 126%) than the Walker Bay Whale Sanctuary (LRT, χ^2^ = 16.5, df = 2, p<0.001).

For catsharks and larger shark species only region and habitat significantly affected FO and mean total relative abundance (S3 Table); protection was not significant, despite qualitative differences, such as a high FO of large sharks (*N*. *cepedianus* FO in Betty’s Bay MPA: 15%) and the only observation of *Sphyrna* sp. recorded in the MPAs. Catsharks had a significantly higher mean relative abundance (LRT, χ^2^ = 26.3, df = 2, p<0.001) and FO (LRT, χ^2^ = 45.4, df = 2, p<0.001) on reef (mean MaxN = 3.4, FO = 93%) and kelp sites (mean MaxN = 3.3, FO = 98%), compared to sand sites (mean MaxN = 0.98, FO = 41%) (S3 Table). In contrast, larger sharks had a marginally greater relative abundance (LRT, χ^2^ = 7.132, df = 2, p = 0.028) and FO (LRT, χ^2^ = 6.36, df = 2, p = 0.042) on sand sites (mean MaxN = 0.29, FO = 23%) compared to reef (mean MaxN = 0.22, FO = 19%) and kelp sites (mean MaxN = 0.19, FO = 17%). Protection, region, and habitat were not significant in our batoid FO model (S2 Table). Although batoid FO appears significantly less in kelp habitat, overall habitat did not improve model fit (LRT, χ^2^ = 5.0, df = 2, p = 0.08).

### Chondrichthyan diversity

**Fig 4:**
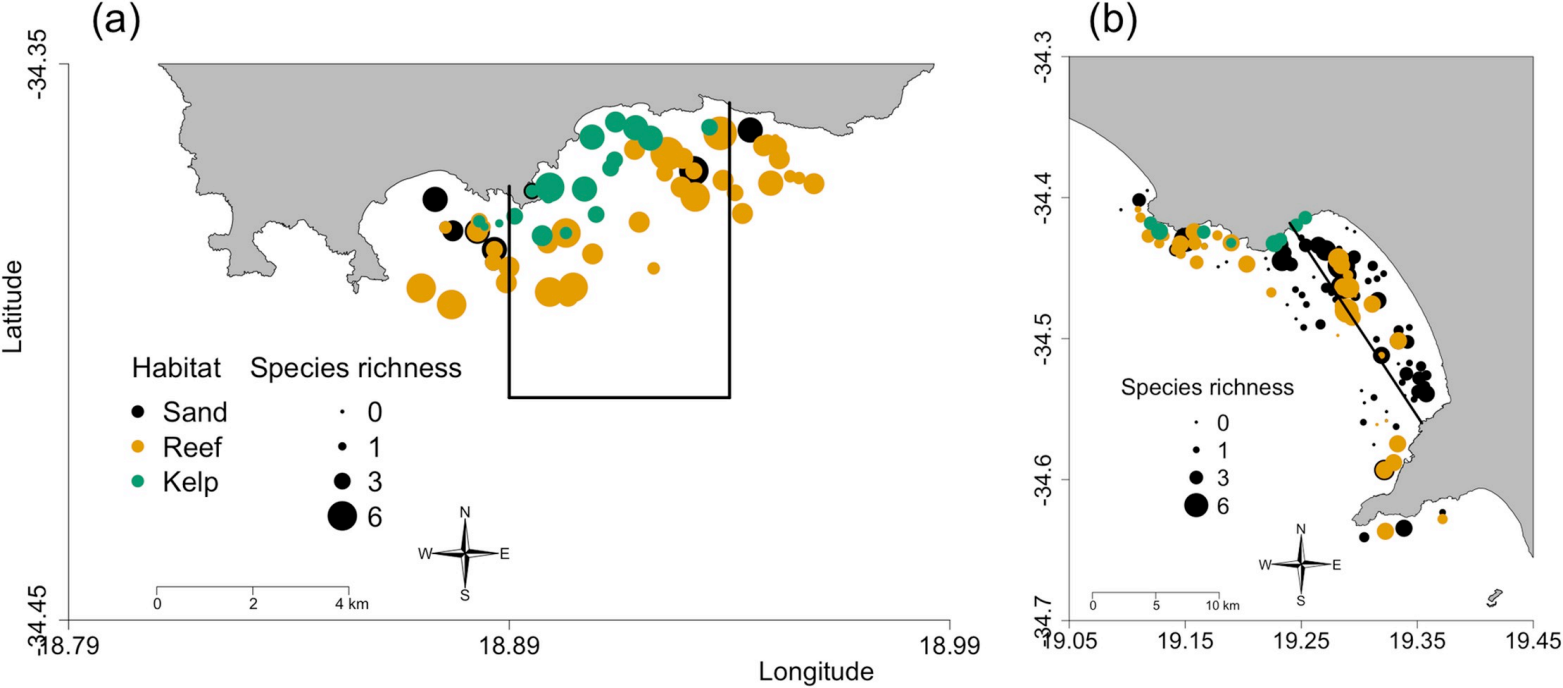
Maps of the study area showing BRUV sites categorized by habitat and species richness in (a) Betty’s Bay and (b) Walker Bay.

Chondrichthyan species richness ranged from 0 to 6 on a single BRUV (mean = 1.9), and overall was significantly higher on reef habitat and lowest in sand habitat (LRT, χ^2^ = 9.7, df = 2, p = 0.008) (S3 Table, Figs 3D and 4). Chondrichthyan species richness was on average greatest in the Betty’s Bay MPA compared to unprotected sites or the Walker Bay Whale Sanctuary, where species richness was the lowest (Figs 3C and 4). As such, the effects of protection on species richness also varied by region. Predicted richness increased by 6% with protection in Betty’s Bay and decreased by 52% with protection in Walker Bay (LRT, χ^2^ = 5.5, df = 1, p = 0.019) (S3 Table). Sites with high species richness were frequent in the Betty’s Bay MPA, and the region in general, while a third of sites in Walker Bay, including those in its MPA (33%), had zero chondrichthyan species. Most chondrichthyan species richness in the Whale Sanctuary was concentrated on a few reef sites (Fig 4).

### Chondrichthyan community composition

**Fig 5:**
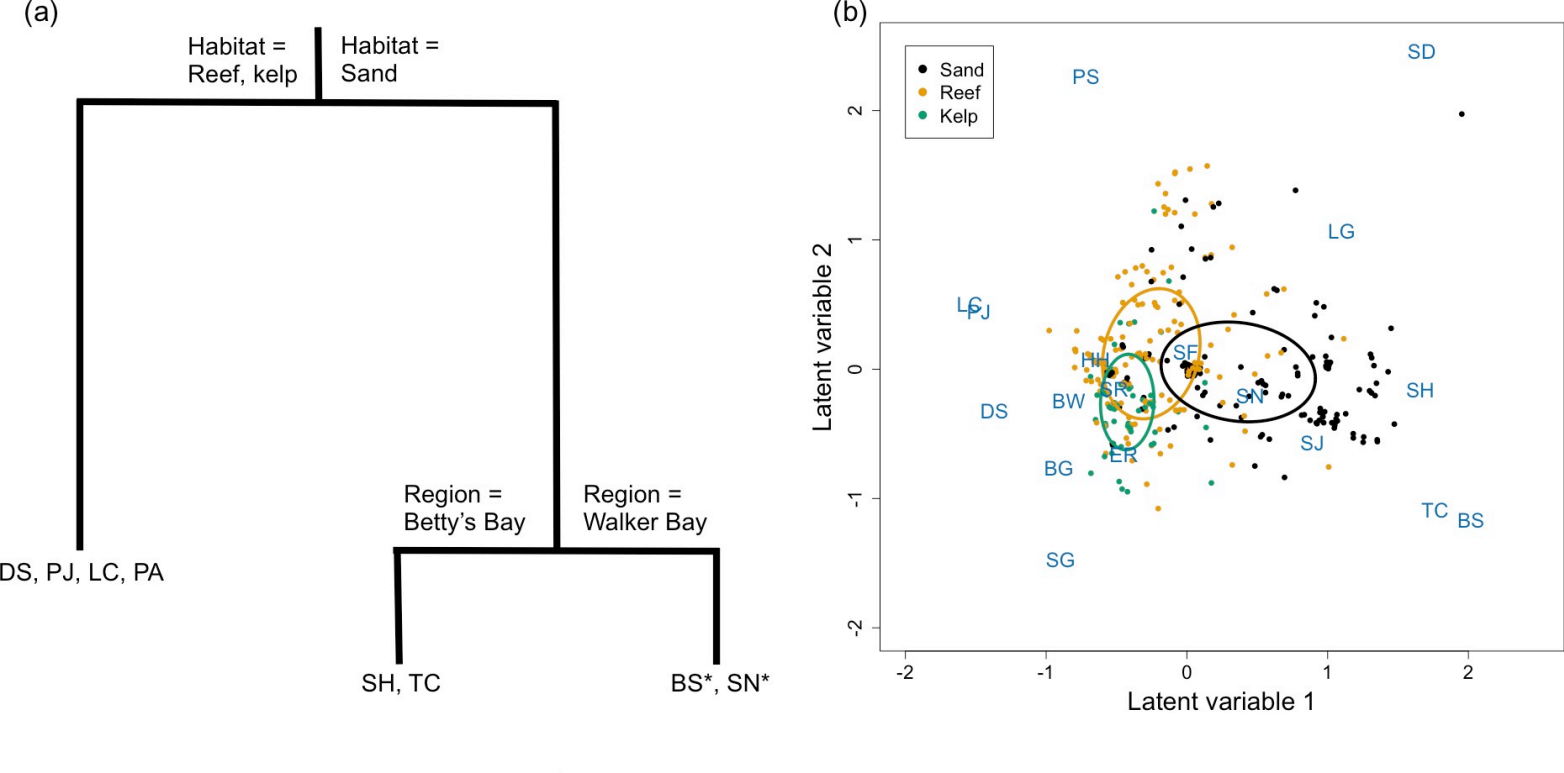
**(a) Multivariate regression tree (MRT) and (b) boral latent variable ordination of the observed chondrichthyan community.** Points are colour-coded by habitat. The ellipses represent 95% confidence intervals around the mean for sites from each habitat. Two-letter species’ codes (explained in Table 1) (a) represent the species with DLI values > 0.15 (except those marked with *, the most important species for that cluster: DLI0.08–0.12), and (b) are positioned to show the relative values for the coefficient for that species on each latent variable axis.

Habitat was the most important variable distinguishing chondrichthyan communities, whereas protection did not distinguish communities on the multivariate regression tree (Fig 5A). Reef and kelp chondrichthyan communities were distinct from those on sand; communities on sand also differed by region (Fig 5A). *Haploblepharus pictus*, *H*. *edwardsii*, *P*. *pantherinum*, and *P*. *africanum* were significant indicators of reef and kelp sites (DLI of 0.65, 0.17, 0.36, 0.46, respectively). In contrast, the tiger catshark *Halaelurus natalensis* and *M*. *mustelus* were significant indicators of sand sites (DLI of 0.14, 0.15, respectively); *Raja straeleni* was also a significant indicator, albeit with a smaller DLI (0.12) for sand habitats, particularly in Walker Bay (Fig 5A). The latent variable ordination confirmed the habitat classification of the multivariate regression tree, showing reef and kelp sites clustering apart from sand sites along the first axis (Fig 5B). The fitted latent variable coefficients placed different chondrichthyan species in each habitat cluster: *H*. *pictus*, *H*. *edwardsii*, *P*. *patherinum*, *P*. *africanum*, *T*. *megalopterus*, and *N*. *cepedianus* had small to negative coefficients for the first latent variable, indicating a higher relative abundance at reef and kelp sites, which were not strongly distinguished (Fig 5B). In contrast, *H*. *natalensis*, *M*. *mustelus*, the St. Joseph shark *Callorhinchus capensis*, the spearnose skate *Rostroraja alba*, *R*. *straeleni*, and the lesser guitarfish *Acroteriobatus annulatus* had positive coefficients for the first latent variable, reflective of their higher relative abundance and occurrence at sand sites (Fig 5B).

### Overlap of habitat and protection

The Walker Bay Whale Sanctuary had a significantly higher frequency of sand sites and a significantly lower frequency of reef sites, while the opposite was true in the Betty’s Bay MPA (*χ*^2^ = 47.185, p<0.001). The unprotected sites in each region had close to the overall average frequency of each habitat type. Kelp sites were rare across all protection levels, but still more frequent in the Betty’s Bay MPA (Fig 4).

## Discussion

Our study found, as predicted, that the chondrichthyan community was dominated by small, endemic, mesopredatory catsharks, while larger shark species and batoids were considerably rarer. The high relative abundance and frequency of occurrence of the endemic catshark species suggests they may have been released from predation, as likely occurs for mesopredatory sharks at isolated, protected tropical reefs [41,42], and that they are currently not heavily threatened by anthropogenic disturbances. However, the population trends of these catsharks are uncertain, and the rarity of other chondrichthyans suggest anthropogenic threats have impacted this region. In particular, the batoid species *R*. *alba* and *R*. *straeleni* are likely threatened along the Cape Whale Coast. Although the former species is not endemic to South Africa, it is endangered globally and its large size makes it vulnerable to extinction [43]. The endemic *R*. *straeleni* is data deficient, but taken by trawls in unknown quantities for human consumption and as bycatch [44].

Larger shark species were seen only infrequently on our BRUVs, which could reflect the impact of fishing, as these sharks, particularly *C*. *brachyurus*, *G*. *galeorhinus* and *M*. *mustelus*, are targeted along the coast [20]. Fisheries pressure has recently increased on *C*. *brachyurus* and it is now being targeted inshore (M. McCord, pers. obs.). The low occurrence of large species is not likely an artifact of selectivity, as size selectivity is minimal for BRUVs [45]. In fact, BRUVs in the Stilbaai MPA ~300 km east of our sites, but with a similar ecosystem, captured a diversity of fishes, including a high relative abundance of larger sharks compared to mesopredators [25]. Notably, despite a great white shark *Carcharodon carcharias* hotspot at Gansbaai, an area near our sites in Walker Bay, we detected no individuals of this species, although sampling in False Bay, South Africa to the west, as well as in Australia suggests BRUVs can be effective at detecting them [46,47]. The endemic *T*. *megalopterus* was also incredibly rare on our BRUVs despite being frequent in the limited catch data. This species has a limited bathymetric range, including a preference for the shallower waters where our BRUVs were deployed, suggesting a low relative abundance and possibly threatened status in the region [48]. The catch data do provide hope for the abundance of some larger sharks, including *T*. *megalopterus* and *N*. *cepedianus*, in the region and corroborate the dominance of small mesopredators in the chondrichthyan community.

Habitat had the strongest influence on chondrichthyan frequency of occurrence, relative abundance, and species richness, all of which were significantly greater in rocky reef and kelp habitat than in sand habitat. The former, in particular, had the strongest effect on chondrichthyan community structure, likely due to its habitat complexity and higher prey biomass. However, both our multivariate analyses identified a distinct community of species found predominantly on the sand habitats, including two data deficient endemics (*R*. *straeleni* and *H*. *natalensis*) and the endemic *C*. *capensis*. These findings align with those of related BRUV studies: a study of tropical sharks on the Great Barrier Reef found proximity to reefs with high coral cover to be the most important factor structuring shark communities and a factor contributing to MPA success [12], while a previous analysis of the fish community in the Betty’s Bay MPA found habitat affected the diversity and species composition while protection had no effect [27]. However, in cases where fishing has had large impacts on populations of sharks and their prey, MPA presence can explain the most variation in shark abundance and community composition compared to environmental and habitat variables alone [49,50]. Our expanded analysis of chondrichthyan abundance and richness did find a limited positive effect of protection in Betty’s Bay not previously detected [27]. Our results also confirm the differences in chondrichthyan community composition between reef and sand sites suggested in False Bay, where *H*. *pictus*, *H*. *edwardsii*, *P*. *patherinum*, and *P*. *africanum* were also found on reef sites and *M*. *mustelus*, *H*. *natalensis* and *G*. *galeus* on sand [46].

We found that, independent of habitat, protection had limited, even negative effects on chondrichthyan relative abundance and species richness, and no effect on community composition. The apparent preference of the endemic catshark community for reef and kelp habitat explains the high diversity and abundance of chondrichthyans within the Betty’s Bay MPA, and in the Betty’s Bay region in general, with its concentration of high quality habitat. Because sand habitat, which had a distinct chondrichthyan community, dominated the Walker Bay Whale Sanctuary, its species richness, frequency of occurrence, and relative abundance of chondrichthyans was low even when compared to unprotected sites nearby. Thus, even though the Betty’s Bay MPA is the smaller of the two protected areas, it has higher potential to protect endemic catshark diversity, should the limited threats they face worsen, especially given the recent re-proclamation of the MPA in South Africa, which outlaws shore angling within MPA boundaries [51]. Additionally, the Betty’s Bay MPA likely plays a role in protecting critical habitat from coastal development, considering Betty’s Bay popularity as a vacation spot near the population centers of Cape Town and Hermanus. South Africa’s endemic catsharks may not be currently threatened, but their abundance in the region is supported by the quality of habitat located in Betty’s Bay, indicating even small MPAs could play a role in protecting coastal ecosystems and endemic species. Spatial protection has had strong effects on resident populations of more threatened sharks on tropical reefs [49,50]; thus MPAs will likely play an important conservation role for endemic South African species should fishing pressure or habitat degradation intensify.

Protection had an even more limited effect on the larger shark species than the catsharks, as these species would likely have regular movements out of either MPA’s boundaries [13,52]. Although we observed a high frequency of *N*. *cepedianus* in the Betty’s Bay MPA, the extent to which this MPA can protect this species depends on its currently unknown movement ecology in the region. The Walker Bay Whale Sanctuary might serve as nursery habitat for the vulnerable *M*. *mustelus*, the largest shark we frequently observed, since it appeared to be smaller in the MPA (G. Osgood, pers. obs.) and is known to show strong residency to shallow bays [24]. Larger, more mobile shark species often show higher residency as juveniles and can benefit from MPAs that protect nursery habitat [53]. Adult *M*. *mustelus* also frequent shallow sandbanks for foraging and predation avoidance [24], and this could explain the relatively high abundance of larger shark species on sand in our study compared to other habitats. However, the overall rarity of large sharks on our BRUVs still suggests these MPAs, even when designed to protect whales, may be too small compared to the home ranges of these larger sharks to effectively curtail fisheries and anthropogenic effects [52]. Ultimately, MPAs cannot replace effective fisheries management for these larger, more mobile species [8].

Marine protected areas, especially no-take zones, have shown preliminary success in other parts of the world in reducing the declines of shark species [5,6,12], but MPAs globally still do not effectively protect chondrichthyan diversity [6]. There is some hope for effective chondrichthyan conservation in South Africa if improved MPA design and enforcement can protect critical habitat, although the smaller, less mobile species that would benefit most are the most abundant in the region’s coastal habitat. Protected areas could benefit more of the threatened species on the Cape Whale Coast if they were expanded to include more area around critical habitats and were enforced in tandem with improved fisheries regulations. Community led co-management initiatives to marine conservation that would engage people with regulations to ensure their enforcement and success would also be beneficial; combining spatial-based management with such community-based fisheries regulations will be an important way forward for marine conservation in developing countries [23].

Ultimately, to succeed in conserving chondrichthyan evolutionary diversity globally, we must understand patterns of chondrichthyan endemism, and implement, enforce and monitor the success of conservation measures to protect it. Non-invasive techniques like BRUVs, which can capture the more elusive, endemic diversity that make developing countries important international targets for conservation, could play an important role in this regard, but to date few BRUV studies have been conducted in these countries. BRUV research carried out in Raja Ampat, Indonesia, similarly showed that the strength of regulations can be more important for shark conservation than MPA size alone [54], but more focus is needed on imperiled endemic diversity. To help establish and monitor conservation measures, as well as improve knowledge of their endemic species, BRUVs programs should be expanded both in South Africa, as the country prepares to increase its MPA coverage to 5% of its exclusive economic zone [55], and in other developing countries [14]. Even small MPAs protecting critical habitat could benefit unique endemic diversity, especially when placed in a network, but they need to be monitored [56,57]. By establishing a baseline, our study represents a step forward in establishing systematic BRUV monitoring of MPAs for endemic chondrichthyan diversity in the developing world.

## Supporting information

S1 Fig Species accumulation curve for (a) all BRUVs in Walker Bay and Betty’s Bay, (b) BRUVs in Betty’s Bay, and (c) BRUVs in Walker Bay.
(TIF)

S1 Table Number of samples in each region and protection level each year in each season (winter: June-November; summer: December-May). (DOCX)

S2 Table Chondrichthyan records in Rock and Surf Super Pro League (2008–2018) and Ocean Research Institute of South Africa tagging databases (2012–2018) from Betty’s Bay and from the South African Shark Conservancy shore and boat fishing databases (2010–2018) in Walker Bay, South Africa. (DOCX)

S3 Table Relative abundance, species richness, frequency of occurrence (FO) model coefficients, with p-values based on Wald’s test shown in brackets, for each fixed effect in generalized linear mixed models (GLMMs), with a baseline of protected, Walker Bay, sand, winter, 2016, and low visibility. (DOCX)

S4 Table Coordinates in decimal degrees of sampling sites from Betty’s Bay (BB) and from Walker Bay (A-G, Y), South Africa. (DOCX)
