# Supplementary figures and images for: Using baited remote underwater videos (BRUVs) to characterize chondrichthyan communities in a global biodiversity hotspot
Source: PLoS One. 2019 Dec 4;14(12):e0225859. doi: 10.1371/journal.pone.0225859 (PMC6892530; doi:10.1371/journal.pone.0225859)

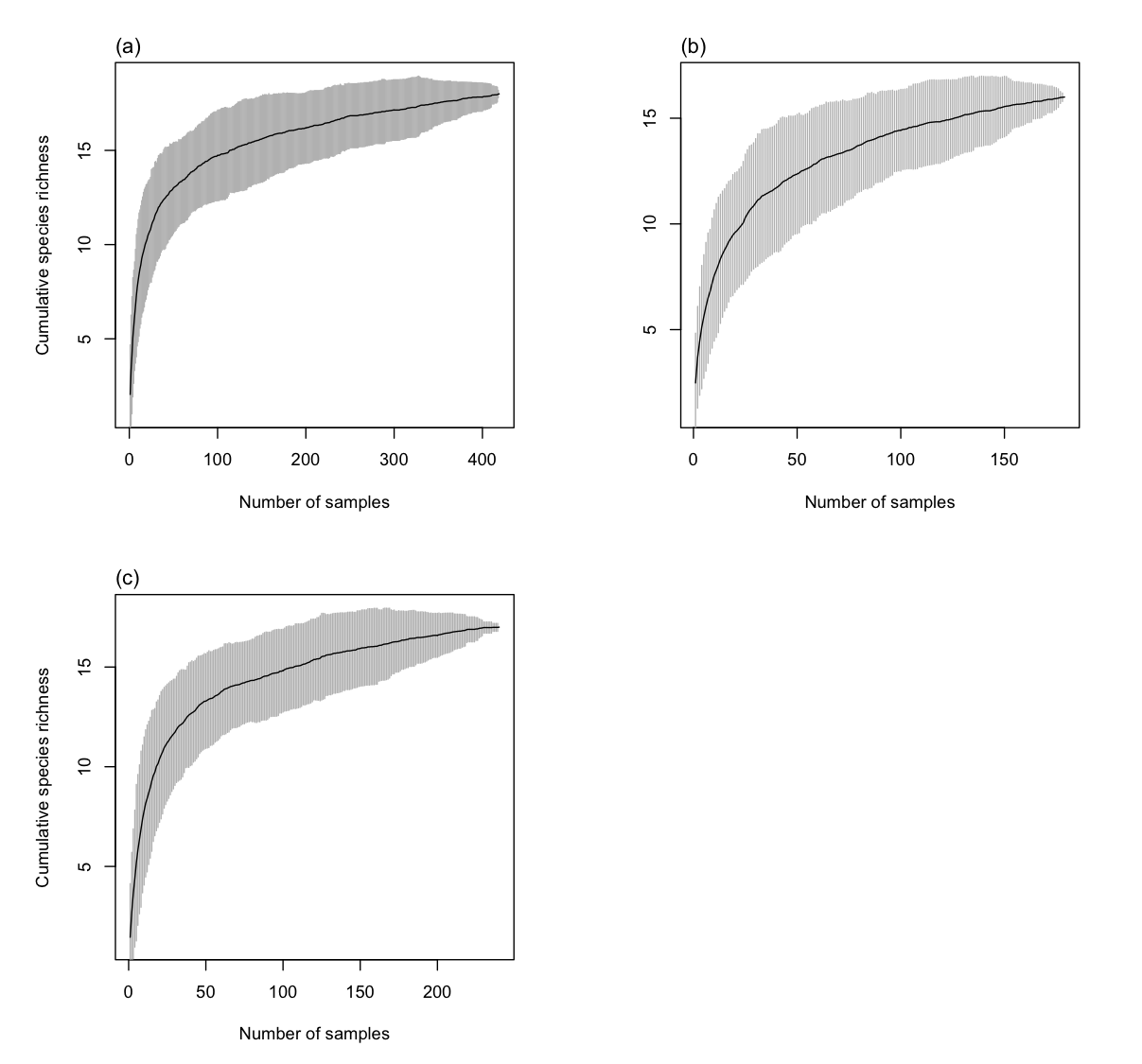

Supplement: S1 Fig — Species accumulation curve for (a) all BRUVs in Walker Bay and Betty’s Bay, (b) BRUVs in Betty’s Bay, and (c) BRUVs in Walker Bay. (TIF) [file pone.0225859.s001.tif]
