# Supplementary material for: Using baited remote underwater videos (BRUVs) to characterize chondrichthyan communities in a global biodiversity hotspot
Source: PLoS One. 2019 Dec 4;14(12):e0225859. doi: 10.1371/journal.pone.0225859 (PMC6892530; doi:10.1371/journal.pone.0225859)
Supplement: S1 Table — (DOCX) [file pone.0225859.s002.docx]

**S1 Table. Number of samples in each region and protection level each year in each season** (winter: June-November; summer: December-May).

|  |  | 2016 | | 2017 | | 2018 | | Total |
| --- | --- | --- | --- | --- | --- | --- | --- | --- |
| Region | Protection | Winter | Summer | Winter | Summer | Winter | Summer |  |
| Walker Bay | Unprotected | 56 | 0 | 18 | 33 | 2 | 22 | 131 |
|  | Protected | 0 | 0 | 9 | 63 | 12 | 25 | 109 |
| Betty's Bay | Unprotected | 0 | 0 | 25 | 29 | 21 | 19 | 94 |
|  | Protected | 0 | 0 | 25 | 27 | 18 | 15 | 85 |
