# Supplementary material for: Using baited remote underwater videos (BRUVs) to characterize chondrichthyan communities in a global biodiversity hotspot
Source: PLoS One. 2019 Dec 4;14(12):e0225859. doi: 10.1371/journal.pone.0225859 (PMC6892530; doi:10.1371/journal.pone.0225859)
Supplement: S2 Table — (DOCX) [file pone.0225859.s003.docx]

**S2 Table. Chondrichthyan records in Rock and Surf Super Pro League (2008-2018) and Ocean Research Institute of South Africa tagging databases (2012-2018) from Betty’s Bay and from the South African Shark Conservancy shore and boat fishing databases (2010-2018) in Walker Bay, South Africa.**

| Species | RASSPL | ORI | SASC | |
| --- | --- | --- | --- | --- |
|  |  |  | Shore-based | Total |
| Scyliorhinidae |  |  |  |  |
| *Haploblepharus pictus* |  |  | 650 | 851 |
| *H. edwardsii* |  |  | 65 | 209 |
| *H. fuscus* |  |  | 12 | 23 |
| *Poroderma africanum* | 13 |  | 200 | 373 |
| *P. pantherinum* |  |  | 203 | 267 |
| total* | 239 |  | 1130 | 1723 |
| Triakidae |  |  |  |  |
| *Triakis megalopterus* | 24 | 166 | 6 | 12 |
| *Mustelus mustelus* | 4 | 9 | 1 | 28 |
| *Galeorhinus galeus* |  |  |  | 28 |
| total* | 70 | 177** | 7 | 68 |
| Other sharks |  |  |  |  |
| *Notorynchus cepedianus* |  | 64 |  | 29 |
| *Carcharias taurus* |  | 1 |  | 2 |
| *Sphyrna zygaena* |  |  |  | 2 |
| *S. lewini* |  |  |  | 2 |
| *Carcharhinus brachyurus* |  |  |  | 7 |
| Batoidea |  |  |  |  |
| *Raja straeleni* |  | 1 | 1 | 13 |
| *Rostroraja alba* |  |  |  | 2 |
| *Acroteriobatus annulatus* |  |  |  | 1 |
| total |  | 1 |  | 16 |
| Holocephali |  |  |  |  |
| *Callorhinchus capensis* | 1 |  |  | 1 |
| Overall total chondrichthyans | 310 | 243 | 1138 | 1850 |

*Includes individuals not identified beyond family, if present

**Two records were identified as Hardnosed Smooth-hound (*Mustelus mosis*) despite this species’ distribution not including Betty’s Bay, South Africa and so are treated as unidentified Triakidae here.
