# Supplementary material for: Using baited remote underwater videos (BRUVs) to characterize chondrichthyan communities in a global biodiversity hotspot
Source: PLoS One. 2019 Dec 4;14(12):e0225859. doi: 10.1371/journal.pone.0225859 (PMC6892530; doi:10.1371/journal.pone.0225859)
Supplement: S3 Table — (DOCX) [file pone.0225859.s004.docx]

**S3 Table.** Relative abundance, species richness, frequency of occurrence (FO) model coefficients, with p-values based on Wald’s test shown in brackets, for each fixed effect in generalized linear mixed models (GLMMs), with a baseline of protected, Walker Bay, sand, winter, 2016, and low visibility.

|  | **Protection: Unprotected** | **Region:**  **Betty’s Bay** | **Protection-**  **Region interaction** | **Habitat: Reef** | **Habitat: Kelp** | **Depth** | **Water temp.** | **Year: 2017** | **Year: 2018** | **Sine time** | **Cosine time** | **Visibility (low vs high)** |
| --- | --- | --- | --- | --- | --- | --- | --- | --- | --- | --- | --- | --- |
| *FO* |  |  |  |  |  |  |  |  |  |  |  |  |
| total | 0.39 (0.59) | 1.35 (0.034)* | NA | 1.21 (0.013)* | 12.2 (0.91) | -0.024 (0.31) | -0.02 (0.86) | 0.97 (0.21) | 0.77 (0.37) | -0.24 (0.56) | 1.06 (0.003)* | 0.98 (0.02)* |
| catsharks | -0.53 (0.44) | 2.35 (<0.001)* | NA | 2.16 (<0.001)* | 3.63 (0.003)* | 0.004 (0.86) | -0.016 (0.87) | 0.52 (0.45) | 0.83 (0.30) | 0.065 (0.87) | 0.52 (0.080) | 1.05 (0.011)* |
| large sharks | -0.47 (0.23) | 1.01 (0.016) | NA | -0.67 (0.067) | -1.23 (0.020)* | -0.026 (0.14) | -0.11 (0.21) | -1.60 (0.011)* | -2.21 (0.0016)* | -0.72 (0.017)* | 0.42 (0.058) | 0.79 (0.05) |
| batoids | -0.07 (0.86) | -0.23 (0.60) | NA | -0.44 (0.25) | -1.39 (0.032)* | -0.005 (0.76) | 0.11 (0.23) | 0.11 (0.85) | -0.22 (0.75) | -0.40 (0.18) | 0.66 (0.0037)* | 0.82 (0.032) |
| *Relative abundance* |  |  |  |  |  |  |  |  |  |  |  |  |
| total | 0.70 (0.035)* | 0.81 (<0.001)* | -0.83 (0.023)* | 0.46 (<0.001)* | 0.49 (0.001)* | 0.005 (0.31) | -0.033 (0.14) | 0.029 (0.85) | 0.15 (0.37) | -0.18 (0.008)* | 0.13 (0.012)* | 0.35 (0.002)* |
| catsharks | -0.023 (0.87) | 0.68 (<0.001)* | NA | 0.71 (<0.001)* | 0.82 (<0.001)* | 0.009 (0.10) | -0.028 (0.25) | 0.011 (0.95) | 0.21 (0.25) | -0.14 (0.056) | 0.099 (0.081) | 0.31 (0.017)* |
| large sharks | -0.096 (0.75) | 0.86 (0.012)* | NA | -0.56 (0.046)* | -1.01 (0.015)* | -0.024 (0.090) | -0.10 (0.16) | -1.22 (0.015)* | -1.73 (0.0016)* | -0.56 (0.024)* | 0.29 (0.085) | 0.56 (0.062) |
| *Species richness* | 0.73 (0.023)* | 0.83 (<0.001)* | -0.80 (0.019)* | 0.32 (0.002)* | 0.22 (0.13) | 0.0015 (0.75) | -0.017 (0.50) | 0.035 (0.84) | 0.084 (0.67) | -0.21 (0.008)* | 0.13 (0.028)* | 0.31 (0.009)* |

*Statistically significant coefficients
