# Supplementary material for: Using baited remote underwater videos (BRUVs) to characterize chondrichthyan communities in a global biodiversity hotspot
Source: PLoS One. 2019 Dec 4;14(12):e0225859. doi: 10.1371/journal.pone.0225859 (PMC6892530; doi:10.1371/journal.pone.0225859)
Supplement: S4 Table — (DOCX) [file pone.0225859.s005.docx]

**S4 Table. Coordinates in decimal degrees of sampling sites from Betty’s Bay (BB) and from Walker Bay (A-G, Y), South Africa.**

| Area | Station | Latitude | Longitude |
| --- | --- | --- | --- |
| A | A2 | -34.39493 | 19.11724 |
| A | A3 | -34.4017 | 19.11019 |
| A | A4 | -34.4086 | 19.09452 |
| A | A5 | -34.40832 | 19.10923 |
| A | A6 | -34.41415 | 19.11148 |
| A | A8 | -34.43242 | 19.1275 |
| A | A9B | -34.4272 | 19.1321 |
| A | A10B | -34.4142 | 19.1115 |
| A | A11 | -34.4399 | 19.1461 |
| A | A12 | -34.43691 | 19.1419 |
| A | A13 | -34.43293 | 19.14573 |
| A | A14 | -34.429873 | 19.15054 |
| A | A15 | -34.43239 | 19.15821 |
| A | A16 | -34.42443 | 19.16587 |
| A | A17 | -34.42417 | 19.15755 |
| A | A18 | -34.4324 | 19.1275 |
| A | A19 | -34.42394 | 19.12781 |
| A | A20 | -34.41812 | 19.12012 |
| A | A21 | -34.4272 | 19.1182 |
| B | B1 | -34.4268 | 19.17771 |
| B | B2 | -34.4345 | 19.16653 |
| B | B4 | -34.4459 | 19.15983 |
| B | B7 | -34.44906 | 19.17839 |
| B | B8 | -34.43208 | 19.18955 |
| B | B12A | -34.44311 | 19.21939 |
| B | B13 | -34.44716 | 19.20303 |
| B | B16 | -34.44481 | 19.2334 |
| B | B17 | -34.43367 | 19.23161 |
| B | B18 | -34.43914 | 19.23576 |
| B | B19 | -34.44735 | 19.24121 |
| C | C1 | -34.4652 | 19.24499 |
| C | C1A | -34.4508 | 19.2217 |
| C | C2 | -34.476 | 19.2379 |
| C | C3 | -34.46727 | 19.22419 |
| C | C4 | -34.46905 | 19.25068 |
| C | C4A | -34.4483 | 19.2019 |
| C | C6A | -34.4456 | 19.1856 |
| C | C7 | -34.47578 | 19.25442 |
| C | C11 | -34.486 | 19.2455 |
| C | C12 | -34.4921 | 19.2521 |
| D | D1 | -34.4977 | 19.2815 |
| D | D9 | -34.54555 | 19.304416 |
| D | D10 | -34.55933 | 19.30357 |
| D | D11 | -34.57521 | 19.3126 |
| D | D12 | -34.59321 | 19.32164 |
| D | D13 | -34.57463 | 19.3331 |
| D | D14 | -34.58799 | 19.32969 |
| D | D15 | -34.56244 | 19.33177 |
| D | D16 | -34.5518 | 19.32353 |
| D | D17 | -34.54177 | 19.3127 |
| D | D18 | -34.55826 | 19.3234 |
| D | D19 | -34.56097 | 19.31528 |
| D | D20 | -34.53692 | 19.30196 |
| E | E1 | -34.50236008 | 19.34161501 |
| E | E2 | -34.492088 | 19.343179 |
| E | E3 | -34.517287 | 19.34328 |
| E | E4 | -34.494141 | 19.333903 |
| E | E5 | -34.47300985 | 19.31605562 |
| E | E6 | -34.531091 | 19.33705 |
| E | E7 | -34.524958 | 19.340608 |
| E | E8 | -34.54043 | 19.339306 |
| E | E9 | -34.543079 | 19.347299 |
| E | E10 | -34.537572 | 19.350699 |
| E | E11 | -34.539124 | 19.357785 |
| E | E12 | -34.47555567 | 19.31112971 |
| E | E13 | -34.51967 | 19.35329 |
| E | E14 | -34.526048 | 19.357881 |
| E | E15 | -34.528132 | 19.351615 |
| E | E16 | -34.53363 | 19.356721 |
| E | E17 | -34.5004897 | 19.31484098 |
| E | E18 | -34.5118795 | 19.31919772 |
| E | E19 | -34.517886 | 19.333751 |
| E | E20 | -34.50156 | 19.33372 |
| F | F1 | -34.42173 | 19.28935 |
| F | F2 | -34.43382 | 19.25385 |
| F | F3 | -34.43379 | 19.26473 |
| F | F5 | -34.43749 | 19.27114 |
| F | F6 | -34.4364 | 19.28265 |
| F | F7 | -34.4640556 | 19.29125521 |
| F | F8 | -34.44806 | 19.28461 |
| F | F9 | -34.46970016 | 19.29656082 |
| F | F10 | -34.45506082 | 19.29033436 |
| F | F11 | -34.45753 | 19.31536 |
| F | F12 | -34.453922 | 19.321057 |
| F | F13 | -34.4484 | 19.311602 |
| F | F14 | -34.44221 | 19.29547 |
| F | F15 | -34.42418 | 19.29604 |
| F | F17 | -34.4433 | 19.28204 |
| F | F18 | -34.45914 | 19.30769 |
| F | F19 | -34.42852 | 19.25 |
| F | F20 | -34.46332325 | 19.28584843 |
| G | G1 | -34.6409 | 19.3043 |
| G | G2 | -34.636668 | 19.322543 |
| G | G3 | -34.634512 | 19.338552 |
| G | G5 | -34.628033 | 19.371857 |
| G | G6 | -34.623233 | 19.371857 |
| F | Kelp1 | -34.414238 | 19.253473 |
| F | Kelp2 | -34.41966 | 19.245623 |
| B | Kelp3 | -34.429718 | 19.23203 |
| B | Kelp4 | -34.432568 | 19.226693 |
| Y | Y1 | -34.4888 | 19.2984 |
| Y | Y2 | -34.4849 | 19.2939 |
| Y | Y3 | -34.4802 | 19.2892 |
| Y | Y4 | -34.47661 | 19.2847 |
| Y | Y5 | -34.4723 | 19.2797 |
| Y | Y6 | -34.46748 | 19.27606 |
| Y | Y7 | -34.46399 | 19.27136 |
| Y | Y8 | -34.4899 | 19.2666 |
| Y | Y9 | -34.4564 | 19.2608 |
| BB | BB10 | -34.3725 | 18.90708 |
| BB | BB12 | -34.36045 | 18.91412 |
| BB | BB16 | -34.36417 | 18.94855 |
| BB | BB17 | -34.37393 | 18.9322 |
| BB | BB18 | -34.38651 | 18.88993 |
| BB | BB24 | -34.38337 | 18.88663 |
| BB | BB28 | -34.38935 | 18.88934 |
| BB | BB29 | -34.39327 | 18.87692 |
| BB | BB3 | -34.38089 | 18.89748 |
| BB | BB36 | -34.380017 | 18.8772 |
| BB | BB38 | -34.390166 | 18.9045 |
| BB | BB39 | -34.39027 | 18.90455 |
| BB | BB40 | -34.38676 | 18.92278 |
| BB | BB43 | -34.39031 | 18.87002 |
| BB | BB46 | -34.3783 | 18.88318 |
| BB | BB48 | -34.37707 | 18.90972 |
| BB | BB49 | -34.391767 | 18.90335 |
| BB | BB5 | -34.37286 | 18.89515 |
| BB | BB50 | -34.3804 | 18.90287 |
| BB | BB51 | -34.38567 | 18.8864 |
| BB | BB52 | -34.3794 | 18.875533 |
| BB | BB55 | -34.36696 | 18.92937 |
| BB | BB56 | -34.36334 | 18.92201 |
| BB | BB57 | -34.36148 | 18.91868 |
| BB | BB58 | -34.36725 | 18.913917 |
| BB | BB59 | -34.368731 | 18.913002 |
| BB | BB6 | -34.372186 | 18.899185 |
| BB | BB60 | -34.36536 | 18.91844 |
| BB | BB61 | -34.36965 | 18.92528 |
| BB | BB62 | -34.37092 | 18.93853 |
| BB | BB63 | -34.37254 | 18.9286 |
| BB | BB64 | -34.36139 | 18.93546 |
| BB | BB65 | -34.36492 | 18.95066 |
| BB | BB66 | -34.37021 | 18.95375 |
| BB | BB67 | -34.36473 | 18.94773 |
| BB | BB68 | -34.36248 | 18.93787 |
| BB | BB69 | -34.36189 | 18.94469 |
| BB | BB70 | -34.36338 | 18.95041 |
| BB | BB71 | -34.36705 | 18.9513 |
| BB | BB72 | -34.37144 | 18.94935 |
| BB | BB73 | -34.37154 | 18.95915 |
| BB | BB75 | -34.37689 | 18.94293 |
| BB | BB76 | -34.37053 | 18.95578 |
| BB | BB77 | -34.36616 | 18.92586 |
| BB | BB78 | -34.37215 | 18.92901 |
| BB | BB79 | -34.36923 | 18.93189 |
| BB | BB8 | -34.36316 | 18.90879 |
| BB | BB80 | -34.37845 | 18.9195 |
| BB | BB81 | -34.38416 | 18.90892 |
| BB | BB82 | -34.37739 | 18.89123 |
| BB | BB83 | -34.37867 | 18.88773 |
| BB | BB84 | -34.37927 | 18.88439 |
| BB | BB85 | -34.37436 | 18.87318 |
| BB | BB86 | -34.38008 | 18.88276 |
| BB | BB87 | -34.38219 | 18.89867 |
| BB | BB88 | -34.39102 | 18.89917 |
| BB | BB9 | -34.37398 | 18.89886 |
| BB | BB90 | -34.37318 | 18.94125 |
